# Editorial: Global Education of Health Management

**DOI:** 10.3389/fpubh.2019.00103

**Published:** 2019-04-30

**Authors:** Connie J. Evashwick, William Edson Aaronson

**Affiliations:** ^1^Department of Health Policy and Management, Milken Institute School of Public Health, George Washington University, Washington, DC, United States; ^2^Department of Health Services Administration and Policy, College of Public Health, Temple University, Philadelphia, PA, United States

**Keywords:** education of healthcare executives, health management education, health administration education, health management & policy, global health systems

The purpose of this special issue is to provide insights about how healthcare executives and managers are educated around the world. As globalization becomes the standard for all industries, healthcare executives must be able to manage effectively with populations, financial arrangements, and technologies that cross geographic boundaries. Education of upcoming students and continuing education of working executives must be broad and encompass a global perspective. Students are increasingly eager to study abroad; our educational programs must include opportunities for students to study in other countries and to have the information in advance that is necessary to make the experience meaningful.

Throughout the world, health systems are grappling with the need to deliver high value healthcare and high quality services despite rapidly increasing costs. The need for effective management to achieve these ends is well-documented. However, healthcare management education is nascent or non-existent in many countries, especially low and middle-income countries that could benefit most from educating healthcare managers in the art and science of management and leadership. This special issue strives to provide insights that might guide universities in developing healthcare management programs in their respective countries.

Hahn and Lapetra present an overview of the Global Competency Directory framework that has five main spheres in which healthcare executives should be competent. This framework was developed by the International Hospital Federation's Global Consortium for Healthcare Management (1). Members included associations representing practice, such as the American College of Healthcare Executives. Hahn and Lapetra offer examples of how the framework can be used in various countries, recognizing diversity among nations while creating a basis for shared content and techniques.

West et al. discuss the role of accrediting organizations in promoting competency-based standards for education. Accrediting bodies such as the Association for the Advancement of Collegiate Schools of Business, the Council on Education for Public Health, and the Commission on Accreditation of Healthcare Management Education are engaged in globalizing accreditation standards. Striving to meet the accrediting standards is an effective strategy in developing healthcare management educational programs.

Glandon reports on how an association that represents graduate and undergraduate healthcare management training programs can promote sharing across disciplines, geographies, and careers. Regional and local healthcare management associations such as the Association of University Programs in Health Administration (AUPHA), the European Health Management Association (EHMA), and SHAPE (representing Australia and New Zealand) provide forums for universities and faculty to learn from others in developing curricula to provide effective healthcare management education.

Drilling down from the role of overarching organizations, two articles explain how healthcare management education has evolved within a single country. Tiwari et al. discuss the evolution of healthcare management education in India. Establishing healthcare management education has been a daunting task in a country so large and diverse. Kalang and Thakur describe the current array of educational institutions and programs educating future healthcare executives and speak to the importance of creating curricula that reflect the evolution of the healthcare system. Both articles challenge the match between the health workforce needs of the nation and the workforce that university programs are producing and articulate considerations for adaptions in healthcare management education.

One of the effective approaches to maximize educational resources across countries is to develop university-to-university partnerships. Two articles provide insights into the details of such partnerships and the benefits provided to each of the partners. Leggat et al. describe a long-standing partnership between universities in China and Australia to train healthcare executives. They emphasize similarities in education despite major differences in the healthcare systems of the two countries.

Different types of schools such as public health, public administration and health/medical schools as well as business schools, can provide healthcare management education. Sammut and Ngoye discuss a collaborative educational program between the University of Pennsylvania Wharton School in the USA and the Strathmore University School of Business in Kenya. The focus of the partnership has been on the establishment of healthcare management as a concentration in the Master of Business Administration program at Strathmore.

Counte et al. advocate for health management education to change as health systems evolve. Specifically, they analyze the trends in healthcare financing. Many countries are exploring value-based payment systems designed to increase quality, reduce costs, and improve population health. The authors describe the implications for curriculum revision that recognize the pervasive impact of these new financing mechanisms.

The majority of articles describe healthcare management programs aimed at graduate students. However, healthcare executive education can start with undergraduate programs and must continue as a life-long commitment. Parviainen et al. report on a national mandate to train mid-career clinicians for management and leadership positions in Finland. Two universities describe their different approaches to fulfilling the mandate. Education of healthcare executives is not only for early careerists, but reaches seasoned managers who seek additional knowledge and clinicians who are tapped to assume management roles. Content, skills, and teaching techniques must be adapted for the experienced student who is being groomed for senior leadership positions.

Sikipa et al. champion the education not only of healthcare executives but of governing board members. Among the advantages they delineate of having a well-trained governing body are better evaluations of the performance of healthcare executives and more astute fiscal responsibility. The content useful to train governing board leaders is also relevant to those doing direct management, varying more in degree of detail than essential topics.

Collectively, these articles show the breadth and diversity of healthcare management education. The challenge to the field is to celebrate the breadth but bring cohesion to content that appropriately spans healthcare systems, cultures, health needs, educational systems, and geographies. Understanding the basic landscape of healthcare management education is a place to start international discussion about how to manage ourselves to achieve consistency of high quality, timely education. Worldwide public health depends on the availability of high quality, accessible, affordable, innovative health services, including primary care, hospitals, and health systems. Effective healthcare management education will improve the likelihood of delivering services designed to provide high quality services, reduce costs, and improve the population's health.

## Author Contributions

Both authors conceived the content of the editorial together. CE wrote the first draft. WA edited and added to the draft. Both agreed on the final version.

### Conflict of Interest Statement

The authors declare that the research was conducted in the absence of any commercial or financial relationships that could be construed as a potential conflict of interest.
